# Global Projections of 21^st^ Century Land-Use Changes in Regions Adjacent to Protected Areas

**DOI:** 10.1371/journal.pone.0043714

**Published:** 2012-08-30

**Authors:** Linda J. Beaumont, Daisy Duursma

**Affiliations:** Department of Biological Sciences, Macquarie University, North Ryde, NSW, Australia; University of Hull, United Kingdom

## Abstract

The conservation efficiency of Protected Areas (PA) is influenced by the health and characteristics of the surrounding landscape matrix. Fragmentation of adjacent lands interrupts ecological flows within PAs and will decrease the ability of species to shift their distribution as climate changes. For five periods across the 21^st^ century, we assessed changes to the extent of primary land, secondary land, pasture and crop land projected to occur within 50 km buffers surrounding IUCN-designated PAs. Four scenarios of land-use were obtained from the Land-Use Harmonization Project, developed for the Intergovernmental Panel on Climate Change's Fifth Assessment Report (AR5). The scenarios project the continued decline of primary lands within buffers surrounding PAs. Substantial losses are projected to occur across buffer regions in the tropical forest biomes of Indo-Malayan and the Temperate Broadleaf forests of the Nearctic. A number of buffer regions are projected to have negligible primary land remaining by 2100, including those in the Afrotropic's Tropical/Subtropical Grassland/Savanna/Shrubland. From 2010–2050, secondary land is projected to increase within most buffer regions, although, as with pasture and crops within tropical and temperate forests, projections from the four land-use scenarios may diverge substantially in magnitude and direction of change. These scenarios demonstrate a range of alternate futures, and show that although effective mitigation strategies may reduce pressure on land surrounding PAs, these areas will contain an increasingly heterogeneous matrix of primary and human-modified landscapes. Successful management of buffer regions will be imperative to ensure effectiveness of PAs and to facilitate climate-induced shifts in species ranges.

## Introduction

Over the past ∼540 million years, there have been five periods of mass extinction, where more than three-quarters of species were lost, and trajectories of recent declines in populations suggests that a sixth mass extinction could occur within the next few centuries [1]. Extinctions over the last 500 years have primarily been driven by human actions, with the last 50 years experiencing the most rapid and extensive ecosystem changes during any period of human activity [2].

In 2002, the Convention on Biological Diversity committed Parties to achieve a ‘significant reduction of the current rate of biodiversity loss at the global, regional and national level’ by 2010 (COP 6 Decision VI/26). Over this period, there have been clear increases in conservation efforts [3], [4]; 87% of countries have developed national biodiversity strategies and action plans, and areas designated as protected now number nearly 133,000, covering ∼12% of the terrestrial surface [4]. While these are positive indicators of endeavours to improve biodiversity conservation, at a global level neither the 2010 target nor any of the 21 sub-targets have been achieved [5]. Biodiversity has continued to decline with no indication of a significant global-scale reduction in human-induced pressures on nature [5]. Rather, although there are examples of local successes in achieving conservation targets, indicators of pressure, such as resource consumption, invasive alien species, and overexploitation, have increased [4].

Given this direction, the Strategic Plan for Biodiversity 2011–2020 and the Aichi Biodiversity Targets (http://www.cbd.int/decision/cop) call for urgent action to prevent the loss of biodiversity and ecosystem services, by addressing underlying causes of biodiversity loss and by decreasing direct pressures on biodiversity. Specifically, this agreement calls for the conservation of at least 17% of terrestrial and inland water areas, and 10% of coastal and marine areas by 2020 (Target 11), and the restoration of at least 15% of degraded ecosystems (Target 15).

However, although protected areas (PAs) are, and will remain, the cornerstone of global conservation efforts [6], an increasing human population and standard of living, and demand for multiple ecosystem services, will intensify competition for the land surrounding PAs [7]. PAs constitute parts of larger ecosystems and are dependent on surrounding landscapes to maintain the flow of organisms, water, nutrients and energy [8]. As such, land-use in areas adjacent to their boundaries can reduce the conservation capacity of PAs through a) changes to the effective size of the reserve, b) alterations to ecological flows into and out of reserves, c) loss of crucial habitat outside of reserves and d) increases in the exposure of park boundaries to negative human impacts [6]. Furthermore, climate change will increasingly play an important role in driving ecosystem changes. Palaeoecological data indicates that species' range changes were the norm during previous episodes of climate change [9], and shifts in the ranges of a variety of taxa in response to 20^th^ century warming have been documented [10], [11]. However, the ability of species to successfully disperse through the landscape will become increasingly difficult should 20^th^ century trends in habitat loss, fragmentation and land-use transition continue.

In this study, we aim to identify broad-scale patterns of land-use change within 50 km buffers surrounding the world's protected areas, using decadal projections of land-use transitions from 2010 to 2050 and 2100. Specifically, we assess the proportion of land within these “buffer regions” projected to undergo conversion between primary land, secondary land, pasture, or crop land. While these land-use categories (developed at a scale of 0.5×0.5 degrees latitude/longitude) are both spatially and descriptively coarse, they provide an initial estimate of future habitat fragmentation and the extent to which these regions may, or may not, facilitate species dispersal to or from PAs as the 21^st^ century progresses.

## Methods

### World Database on Protected Areas (WDPA)

The 2010 WDPA (http://www.wdpa.org/) is the most comprehensive dataset on the global distribution of marine and terrestrial PAs. It contains spatial and attribute data for nationally designated (e.g. National Parks, Nature Reserves) and internationally recognized PAs. An area is only accepted for inclusion in the WDPA if it is ‘A clearly defined geographical space, recognized, dedicated and managed, through legal or other effective means, to achieve the long term conservation of nature with associated ecosystem services and cultural values’ [12]. Due to licencing restrictions WDPA does not contain data for England, and there may be parks in other countries that are yet to be incorporated into the database.

### Land-use Projections

In order to assess the impact of human activities on the carbon-climate system, new Earth System Models (ESMs) are being developed for the Fifth Assessment Report of the Intergovernmental Panel on Climate Change [13], [14]. The ESMs required projections of future land-use changes, which were derived from four Integrated Assessment Models (IAMs) of four Representative Concentrations Pathways (RCPs) [14]. The Land-Use Harmonization project was developed to smoothly connect historical reconstructions of land-use changes with projections of future changes [13]. The RCPs span the range of radiative forcing levels for 2100 in the literature, and have been given names reflecting these levels (i.e. 2.6, 4.5, 6.0, 8.5 watts per square metre) [14]. Briefly, RCP2.6 (IAM: IMAGE) is a low greenhouse scenario, with a radiative forcing pathway that peaks at ∼490 CO_2_-equiv (around 2070), and then declines; RCP4.5 (IAM: GCAM, previously referred to as MiniCAM) reaches stabilization without overshoot after 2100 at ∼650 CO_2_-equiv; RCP6.0 (IAM: AIM) also reaches stabilization without overshoot after 2100 but at ∼850 CO_2_-equiv; RCP8.5 (IAM: MESSAGE) exceeds 1,370 CO_2_-equiv in 2100, with a pathway that continues to rise and underlying scenario drivers based on the IPCC A2 scenario [14], [15]. Land-use scenario projections across the four RCPs can vary substantially. To summarise, in RCP8.5 net increases in cultivated land occur in developing countries, due to increasing population, while forest cover declines. RCP6 incorporates mitigation actions late in the century. In this pathway cropland expands while the total extent of forest cover remains constant. RCP4.5 assumes that global greenhouse gas emissions pricing limits carbon emissions, resulting in the preservation of terrestrial carbon in forests. Due to afforestation the extent of agricultural land declines and the total forested area increases slightly. In RCP2.6 agricultural lands relocate from high- to low-income regions, and bio-energy crops increase [13]. As such, these pathways enable an exploration of the consequences of different adaptation and mitigation strategies, and interactions between drivers of change [16].

We obtained scenarios of 21^st^ century land-use from the Land-Use Harmonization project [13] (http://luh.umd.edu) (i.e. LUHa_u2t1.v1, LUHa_u2t1.v1_image.v1.1, LUH_u2t1.v1_minicam.v1, LUHa_u2t1.v1_aim.v1.1, LUH_u2t1.v1_message.v1, which are based on RCP2.6, RCP4.5, RCP6.0, RCP8.5, respectively). These data comprise annual estimates of fractional land-use for crop, pasture, urban, primary (i.e. natural vegetation with no prior land-use history), and secondary land (i.e. recovering from previous human land-use activity), and underlying land-use transitions (describing changes in land-use such as harvesting trees and establishing or abandoning agricultural land). They are based on historical simulations that begin in 1700, and are at a spatial resolution of 0.5°×0.5°. Whether a given grid cell contains protected areas or not is currently not considered in calculations of land-use transition (L.P. Chini, pers comm. May 2012).

### Buffer development and data extraction

Using ArcGIS v10.0 [17], we extracted all terrestrial PAs with IUCN categories I–VI from WDPA2010. This totalled ∼64,800 PAs. Data were converted from WGS84 to a cylindrical equal areas projection. We used Geospatial Modelling Environment [18] to create a 50 km buffer surrounding each PA. This distance was selected as it approximates the area contained within a grid cell of the Land-Use Harmonization data at low latitudes (i.e., 0.5° latitude x longitude). Further, the habitat within 50 km buffers surrounding PAs in tropical forests has been shown to influence the conservation capacity of PAs [19]. Land within the PAs was masked out, i.e. not included in the buffers.

The topology of some PAs was very complex, consisting of many small polygons. For these, we used ArcGIS's Explode tool to separate multi-part features into their components. Buffers were placed around each part, then merged to create a single buffer for that feature. However, approximately 100 reserves (from USA and Europe) remained too complex for buffers, and these were removed from analyses. For consistency with the Land-Use Harmonization data, buffer layers were converted back into a geographic coordinate system (WGS84). In some regions where there are a large number of small PAs, buffers overlapped considerably. Ideally, the boundaries of overlapping buffers would have been dissolved to prevent projections for the same area of land being calculated multiple times. However, dissolving such a large number of polygons was not possible. Instead, we converted a shapefile of all polygons into a binomial raster grid (0 = outside of buffer, 1 = inside buffer). This conversion meant that only those grid cells that had at least 50% of their area covered by a buffer were classified as “1”.

**Figure 1:**
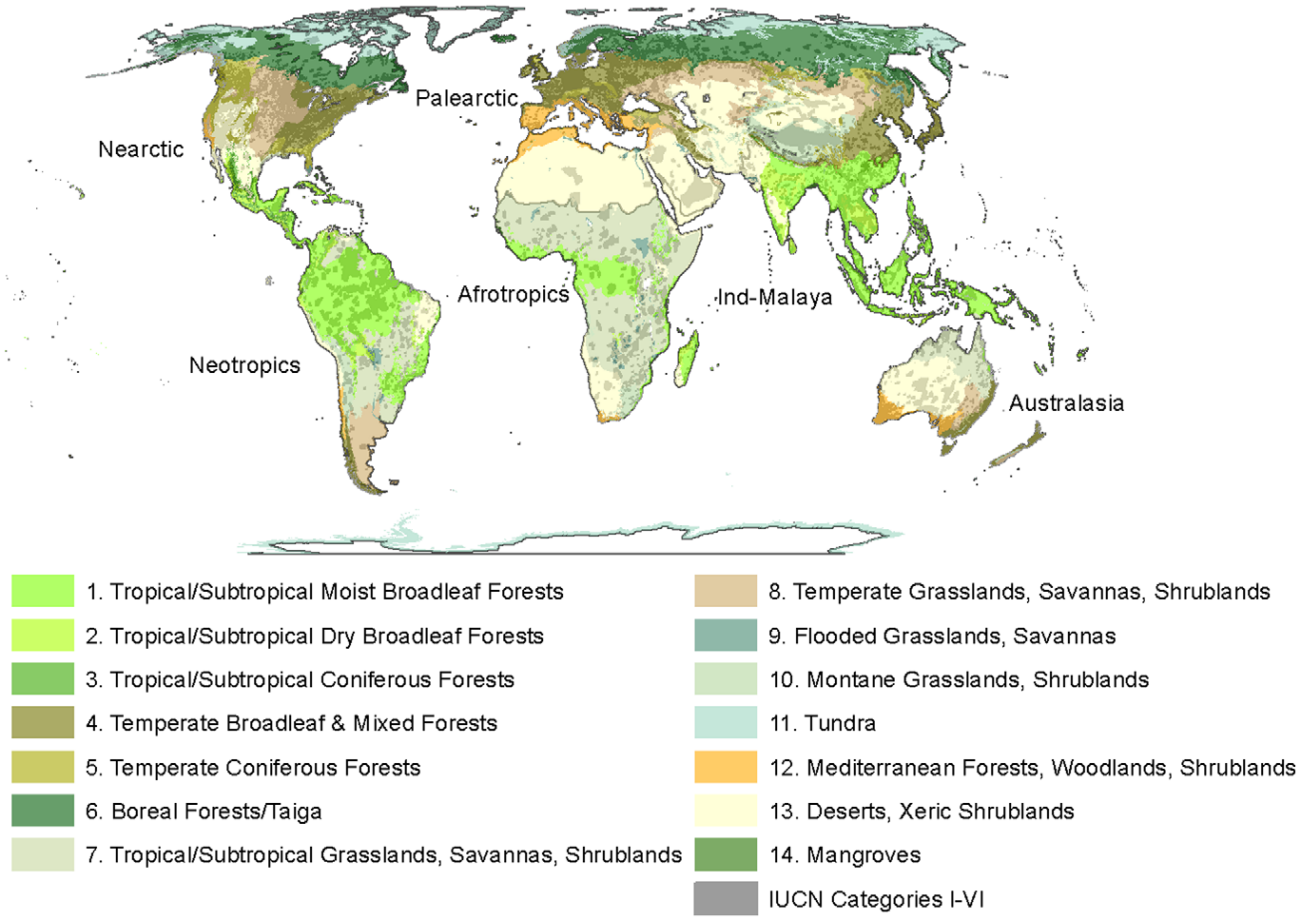
WWF biogeographic realms and biomes, and IUCN Protected Areas (Categories I–VI).

Each buffer grid cell was classified according to the WWF Biome and biogeographic realm [20] within which the centre of the grid cell occurred. Biomes included 1) Tropical/Subtropical Moist Broadleaf Forest, 2) Tropical/Subtropical Dry Broadleaf Forest, 3) Tropical/Subtropical Coniferous Forest, 4) Temperate Broadleaf and Mixed Forest, 5) Temperate Coniferous Forest, 6) Boreal Forest/Taiga, 7) Tropical/Subtropical Grassland, Savanna and Shrubland, 8) Temperate Grassland, Savanna and Shrubland, 9) Flooded Grassland and Savanna, 10) Montane Grassland and Shrubland, 11) Tundra, 12) Mediterranean Forest, Woodland and Shrubland, 13) Desert and Xeric Shrubland, and 14) Mangrove. Two biomes, Lakes and Rock/Ice, were excluded from analysis. The seven biogeographic realms were Nearctic, Neotropic, Palearctic, Afrotropic, Indo-Malaya, Australasia, and Oceania (Figure 1). Due to the small size of landmasses within the Oceanic, this realm was excluded from analyses. This resulted in 56 combinations of realms x biomes, which we refer to as ‘buffer regions’. These were intersected with the four land-use scenarios to calculate the average proportion of primary land, secondary land, pasture and crop land projected to occur for six time periods (2010, 2020, 2030, 2040, 2050 and 2100). Our analyses assume that the current arrangement of PAs remains constant over time.

## Results

### Primary land

**Figure 2:**
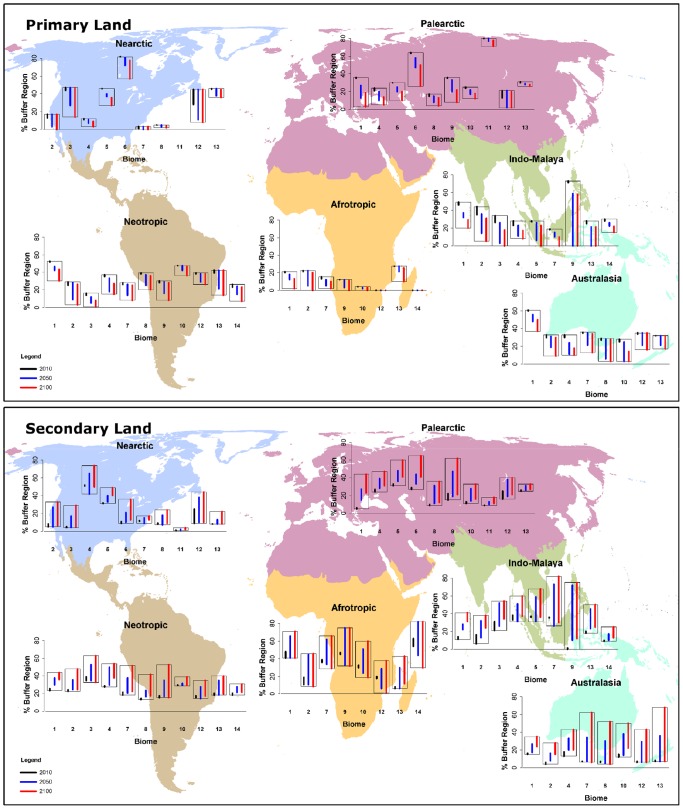
Projections of 21^st^ century land-use within 50 km buffers surrounding protected areas. This figure shows the projected extent of a) primary land and b) secondary land within 50 km buffers surrounding protected areas. Results are summarised for each biome in six biogeographic realms. Projections are for three time periods i) 2010 (black), ii) 2050 (blue) and iii) 2100 (red), and were derived from four scenarios of land-use. The length of the bars in each graph shows the variation among these four scenarios. The biomes are: 1) Tropical/Subtropical Moist Broadleaf Forest, 2) Tropical/Subtropical Dry Broadleaf Forest, 3) Tropical/Subtropical Coniferous Forests, 4) Temperate Broadleaf and Mixed Forest, 5) Temperate Coniferous Forests, 6) Boreal Forests/Taiga, 7) Tropical/Subtropical Grassland, Savanna and Shrubland, 8) Temperate Grassland, Savanna and Shrubland, 9) Flooded Grasslands and Savannas, 10) Montane Grassland and Shrubland, 11) Tundra, 12) Mediterranean Forest, Woodland and Shrubland, 13) Desert and Xeric Shrubland, and 14) Mangroves.

As at 2010, the extent of primary lands across buffer regions was estimated to be between 16% (Afrotropics) to 44% (Indo-Malaya, Nearctic, Neotropics, Figure 2a). Averaged across the four land-use scenarios, primary land is projected to decline by at least 30% within 18 and 40 of the 56 buffer regions by 2050 and 2100, respectively. Buffer regions within the Tropical/Subtropical Dry and Moist Broadleaf Forests, Flooded Grasslands/Savannas and Temperate Broadleaf/Mixed Forest biomes are projected to lose substantial primary land, and by 2100 some regions may have negligible primary land remaining (Table S1). These include buffer regions in the Palearctic Moist Broadleaf Forest (2010: 35–36%, 2100: 3–19%), Afrotropic Grasslands/Savannas/Shrublands (2010: 13–15%; 2100: 2–10%), and the Nearctic Temperate Broadleaf/Mixed Forests (2010: 11–12%; 2100: 3–9%). In contrast, those in Tundra or Desert/Xeric Shrublands are projected to have smaller losses of primary lands.

**Figure 3:**
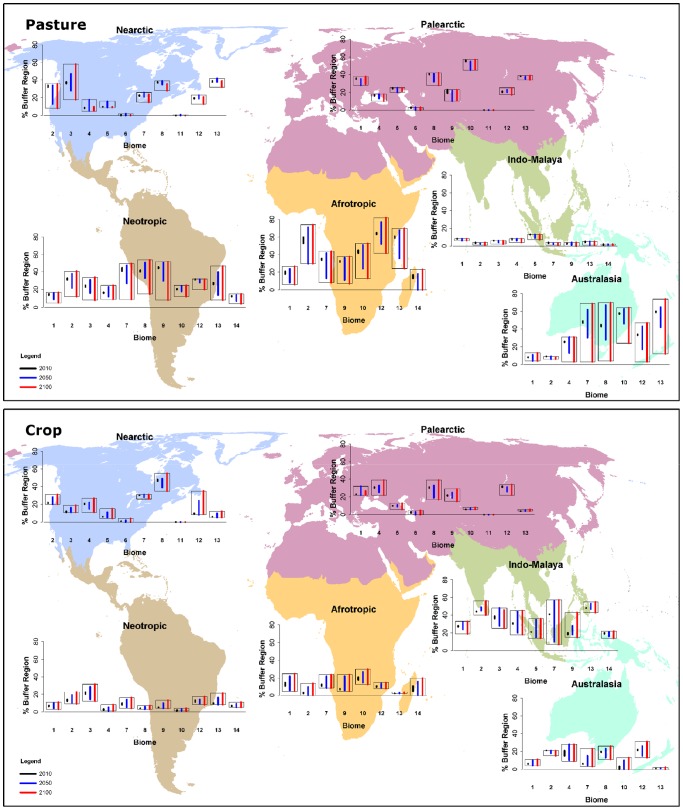
Projections of 21^st^ century land-use within 50 km buffers surrounding protected areas. Similar to Figure 2, this figure shows the projected extent of a) pasture land and b) crop land within 50 km buffers surrounding protected areas.

Greater consistency occurred among projections of the four land-use scenarios for primary land compared with the other land-uses (Figures 2a, b and 3a, b). However, for 2050, between-scenario variability (i.e. largest proportional change minus the smallest proportional change) exceeded 20% for eight of the 56 buffer regions, particularly those in Indo-Malaya. By 2100, between-scenario variability exceeded 20% for an additional seven buffer regions, including the Boreal Forest/Taiga of the Palearctic and Nearctic. Generally, RCP4.5 projected smaller loss of primary land in buffer regions, while RCP8.5 projected greater losses.

Most buffer regions are located within temperate areas. Those in the Temperate Broadleaf/Mixed Forests of Australasia and Palearctic are projected to lose ∼40–70% of primary land by 2100, depending upon the land-use scenario, resulting in 10–18% and 5–14% coverage, respectively. Similarly, the buffer region in the Temperate Forests of the Nearctic may lose 24–78% of primary land, resulting in 3–9% coverage by 2100. Within buffers in the Palearctic Temperate Conifer biome, primary land may decline from 30% (2010) to 10–20% in 2050, and 5–14% by 2100. In the Boreal/Taiga forests of the Nearctic and Palearctic primary land currently covers 82% and 63–64% of buffer regions, respectively. This is projected decline to 57–77% (Nearctic) and 26–50% (Palearctic) by 2100.

More than 50% of primary land within buffer regions of the Moist Broadleaf Forests of Indo-Malay and the Afrotropics is projected to be lost by 2100 (Indo-Malay 2010: 46–49%, 2100: 20–29%; Afrotropics 2010: 20–21%, 2100 2–13%), depending upon the land-use scenario. Buffer regions containing Mediterranean Forests/Woodlands/Scrublands occur in Australasia, Neotropics, Nearctic, and Palearctic. For these regions, primary land is projected to decline by an average 17% (Neotropics) to 45% (Nearctic) by 2100. However, considerable inter-model variability occurs, with declines for 2100 ranging from <1% to 80% in the Nearctic.

Currently, the Flooded Grassland/Savanna biome covers <1% of the earth's terrestrial area, and is present in few (72) of the original ∼64,800 buffers. In the Palearctic, primary land within these buffers is projected to decrease from 35–36% in 2010 to 20–33% by 2050, and 8–22% by 2100.

### Secondary land

Across the six realms, buffer regions were projected to comprise between 9% (Australasia) to 35% (Afrotropics) secondary land as at 2010 (Figure 2b). Across these two realms secondary land is projected to increase to 11–32% and 32–57%, respectively, by 2050, and by 2100 may cover 11–55% and 29–62% of these buffer regions.

Substantial increases are projected for buffer regions in the Tropical/Subtropical Moist and Dry Broadleaf Forests of Indo-Malaya (Moist 2010: 11–14% and 2100: 31–41%; Dry 2010: 6–16% and 2100: 24–38%) (Table S2). Buffer regions in Boreal/Taiga forests are also projected to have substantial expansion of secondary land, primarily in the second half of the century, particularly for the Palaearctic (2010: 27–29%; 2100: 41–65%). In contrast, three of the four land-use scenarios project little change to the extent of secondary land in buffer regions in Temperate forests of the Nearctic (2010: 51–52%, 2100: 50–57%, although RCP4.5 projects 74%).

Considerable between-scenario variability occurs among projections of secondary land. For 35 of the 56 buffer regions, between-scenario variability exceeds 20% by 2100. This is particularly the case for Tropical/Subtropical Grassland/Savanna/Shrubland and Deserts/Xeric Shrublands in Australasia. Scenarios project secondary land to occupy between 6–62% and 7–68% of these two buffer regions, respectively. Similarly, for the buffer region of the Afrotropics Tropical/Subtropical Grassland/Savanna/Shrubland biome projections range from a decrease of 8% (RCP8.5) to an increase of 28% (RCP6) by 2100.

### Pasture lands

Currently, pasture lands occupy between 6–7% (Indo-Malaya) to 42–43% (Australasia) of buffers surrounding PAs (Figure 3a). The largest changes to the extent of pasture across buffer regions is projected for those in the three Grassland/Shrubland biomes, particularly in the Tropical/Subtropical regions. However, alternate scenarios can project changes to occur in different directions. For example, for this buffer region in the Afrotropics pasture lands may increase 9% by 2100 (RCP8.5: 2010: 35%; 2100: 44%) or decrease 26% (RCP6: 2010: 34%; 2100: 8%). Between-scenario variability exceeded 20% across 22 of the 56 buffer regions by 2100 (Table S3). Divergences primarily occur in Australasia, the Neotropics and the Afrotropics. These divergences are particularly large for buffers in the Australasian Tropical, Temperate and Xeric shrublands. In contrast, there was greater concensus across projections for Indo-Malay.

### Crop lands

Currently, crop lands occupy 7–8% (Neotropics) to 32–33% (Indo-Malay) of buffer regions. For most buffer regions these proportions are projected to remain relatively stable between 2010 and 2100 (Figure 3b), and consensus across the four land-use scenarios is generally high. Divergences among the scenarios exceed 20% for only nine of the 56 realm/biome combinations, by 2100 (Table S4). Projections differ most dramatically for buffer regions in Indo-Malay, and for Temperate Grassland/Shrublands in the Palaerctic and Nearctic. For example, for the Palearctic the current extent of crops in buffers is ∼31%, but is projected to decline to 17% (RCP4.5) or increase to 39% (RCP6) by 2100.

## Discussion

Protected areas (PA) have been described as analogous to islands of natural or semi-natural habitat in an ocean of land transformation [21], and this description will become increasingly apt as the century progresses. We have undertaken a coarse estimate of changes to land-use within 50 km buffers surrounding IUCN-designated Protected Areas, projected for the 21^st^ century. PAs are the primary focus of the nature conservation agenda [19], [21], [22], and their coverage of globally significant sites can decrease extinction risks [23]. However, characteristics of the surrounding landscape matrix will impact the conservation capacity of PAs, as well as the ability of species to disperse through these regions as climate changes.

Estimates of land-use during the period 1700–2000 suggests that 42–68% of terrestrial regions were impacted by activities such as crop, pasture and wood harvesting [24]. Wood harvesting and shifting cultivation were responsible for 70–90% of secondary land by 2000, while permanent abandonment and relocation of agricultural lands accounted for the remainder [24]. Presently, primary lands occupy between 16% (Afrotropics) to 44% (Indo-Malaya, Nearctic, Neotropics) of buffer regions surrounding PAs, depending upon the biogeographic realm. Land-use scenarios project decreases to the extent of primary land within the buffer regions of all realms throughout the 21^st^ century, with those in the Temperate Forests of Australasia, Nearctic and Palearctic projected to lose up to 69–78% of primary land by 2100.

Tropical forests within 50 km of PAs in Indo-Malaya have declined substantially since the 1990s, impacting on the capacity of the reserves to conserve species richness [19]. Buffers containing Tropical/Subtropical Moist Broadleaf Forest, Dry Broadleaf Forest, and Coniferous Forest biomes within this realm are projected to lose 24–33%, 18–59% and 7–23% of primary land by 2050, respectively, relative to 2010. These losses may increase to more than 50% by 2100, under some land-use scenarios. Further, while the magnitude of mean annual temperature change has been greatest at higher latitudes, it is equatorial regions, characterised by low inter- and intra-annual variability in temperature, that will experience novel climatic conditions soonest [25]. Combined, the changes in land-use and climate will place increasing pressure on biodiversity within these regions.

Globally, secondary land within buffer zones is projected to increase throughout the century, particularly across buffers in some tropical forest biomes of Australasia and Indo-Malaya. Demand for crop land will depend on the balance between improvements in yield, an increase in the number of crops grown in a season, and increases in agricultural and bioenergy demands [7]. The expansion of crop and pastoral land into natural ecosystems has been a major driver of global biodiversity loss [26], [27]. Projections of the amount of land used for agricultural purposes across the 21^st^ century depend on scenarios of changes to profitability, the value of terrestrial carbon storage and improvements to agricultural productivity. As such, the magnitude and rate of change of alternate land-uses within buffers can vary across the different land-use scenarios. For most buffer regions changes to the extent of agricultural lands are projected to be small for the first half of this century (e.g. within 5%), although greater variability among the scenarios is projected within the tropics. Projections from the four scenarios may diverge further over time, casting considerable uncertainty on future changes to the extent of crop and pasture lands in regions surrounding PAs.

As the century progresses, conservation efforts in landscapes surrounding PAs will be forced to focus on secondary and other human-modified landscapes. In tropical Asia, America and Africa, 60% of areas classified as forest consist of degraded primary and secondary lands, covering an estimated 850 million ha in 2000 [28]. While the predominant use of secondary land in these regions is as fallow within shifting cultivation systems [28] these landscapes, as well as semi-natural and agro-ecosystems, can play an important role in conservation [29]. Comparisons of faunal diversity in tropical secondary forests and old-growth forests suggests that similarity to old-growth forests may increase rapidly in a number of situations, e.g. with secondary forest age, when secondary forests are contiguous with old-growth forests, and after low intensity land-uses [30]. Similarly, appropriately designed agro-ecosystems have demonstrated that both biodiversity and agricultural yield can be maximised without placing higher pressure on remaining primary lands [26], and greater agricultural yields can decrease the land area required to reach a given level of production [27].

However, the conservation value of secondary and other human-modified landscapes remains lower than primary forests [31]. While secondary forests may serve as a ‘safety net’ for tropical biodiversity [32] they are unlikely to conserve many primary forest species, and although there are examples of land-use transitions that have succeeded in increasing both forest cover and agricultural production, these require substantial understanding of the land system [27]. For many regions, the consequences of human impacts are relatively unknown, or are based on a limited number of localised studies [33].

Our results show that over the course of this century the landscape matrix surrounding protected areas may become increasingly fragmented. The categories of land-use we used are spatially and descriptively coarse, and do not assess either the spatial arrangement or size of land-use fragments within each 0.5×0.5° grid cell. As such, while some scenarios may project negligible change to the overall extent of a given land-use within buffer regions, this does not mean that the spatial arrangement of these patches will remain constant. We suggest that similar assessments be repeated over regions of interest as more detailed, fine-resolution projections of land use changes become available. Regardless, the effectiveness of many PAs may be impacted by the resulting isolation, edge effects, and dispersal barriers projected to occur. Fragmentation of the landscape matrix will also present substantial barriers to climate-induced range shifts of species.

Finally, uncertainty in drivers of change can lead to considerable variability in projections of land-use change within some regions. These differences demonstrate that a range of options are available which may lessen the impact of anthropogenic climate change on biodiversity. For example, an increase in global forested lands (including those in the tropics) is possible under certain climate mitigation policies and levels of agricultural productivity [34]: the inclusion of emissions pricing in RCP4.5 is projected to result in the preservation of large stocks of terrestrial carbon in forests, and afforestation of agricultural lands [13]. This suggests cautious optimism that the destruction of primary lands may be reduced, a move that would have clear benefits to global conservation efforts.

## Supporting Information

Table S1 Projections of the fractional extent of primary land across 50 km buffer regions that surround the world's Protected Areas. Projections were derived from four land-use scenarios, and are summarized for each Realm/Biome. The table gives the smallest and largest projection of the four scenarios for 2010, 2050 and 2100. Biomes are 1) Tropical/Subtropical Moist Broadleaf Forest, 2) Tropical/Subtropical Dry Broadleaf Forest, 3) Tropical/Subtropical Coniferous Forests, 4) Temperate Broadleaf and Mixed Forest, 5) Temperate Coniferous Forests, 6) Boreal Forests/Taiga, 7) Tropical/Subtropical Grassland, Savanna and Shrubland, 8) Temperate Grassland, Savanna and Shrubland, 9) Flooded Grasslands and Savannas, 10) Montane Grassland and Shrubland, 11) Tundra, 12) Mediterranean Forest, Woodland and Shrubland, 13) Desert and Xeric Shrubland, and 14) Mangroves.
(DOCX)

Table S2 Projections of the fractional extent of secondary land across 50 km buffer regions that surround the world's Protected Areas. Projections were derived from four land-use scenarios, and are summarized for each Realm/Biome. The table gives the smallest and largest projection of the four scenarios for 2010, 2050 and 2100. See Table S1 for the names of each biome.
(DOCX)

Table S3 Projections of the fractional extent of pasture land across 50 km buffer regions that surround the world's Protected Areas. Projections were derived from four land-use scenarios, and are summarized for each Realm/Biome. The table gives the smallest and largest projection of the four scenarios for 2010, 2050 and 2100. See Table S1 for the names of each biome.
(DOCX)

Table S4 Projections of the fractional extent of crop land across 50 km buffer regions that surround the world's Protected Areas. Projections were derived from four land-use scenarios, and are summarized for each Realm/Biome. The table gives the smallest and largest projection of the four scenarios for 2010, 2050 and 2100. See Table S1 for the names of each biome.
(DOCX)
