# Supplementary material for: Global Projections of 21st Century Land-Use Changes in Regions Adjacent to Protected Areas
Source: PLoS One. 2012 Aug 30;7(8):e43714. doi: 10.1371/journal.pone.0043714 (PMC3431375; doi:10.1371/journal.pone.0043714)
Supplement: Table S1 — Projections of the fractional extent of primary land across 50 km buffer regions that surround the world's Protected Areas. Projections were derived from four land-use scenarios, and are summarized for each Realm/Biome. The table gives the smallest and largest projection of the four scenarios for 2010, 2050 and 2100. Biomes are 1) Tropical/Subtropical Moist Broadleaf Forest, 2) Tropical/Subtropical Dry Broadleaf Forest, 3) Tropical/Subtropical Coniferous Forests, 4) Temperate Broadleaf and Mixed Forest, 5) Temperate Coniferous Forests, 6) Boreal Forests/Taiga, 7) Tropical/Subtropical Grassland, Savanna and Shrubland, 8) Temperate Grassland, Savanna and Shrubland, 9) Flooded Grasslands and Savannas, 10) Montane Grassland and Shrubland, 11) Tundra, 12) Mediterranean Forest, Woodland and Shrubland, 13) Desert and Xeric Shrubland, and 14) Mangroves. (DOCX) [file pone.0043714.s001.docx]

Table S1. Projections of the fractional extent of primary land across 50km buffer regions that surround the world’s Protected Areas. Projections were derived from four land-use scenarios, and are summarized for each Realm/Biome. The table gives the smallest and largest projection of the four scenarios for 2010, 2050 and 2100. Biomes are 1) Tropical/Subtropical Moist Broadleaf Forest, 2) Tropical/Subtropical Dry Broadleaf Forest, 3) Tropical/Subtropical Coniferous Forests, 4) Temperate Broadleaf and Mixed Forest, 5) Temperate Coniferous Forests, 6) Boreal Forests/Taiga, 7) Tropical/Subtropical Grassland, Savanna and Shrubland, 8) Temperate Grassland, Savanna and Shrubland, 9) Flooded Grasslands and Savannas, 10) Montane Grassland and Shrubland, 11) Tundra, 12) Mediterranean Forest, Woodland and Shrubland, 13) Desert and Xeric Shrubland, and 14) Mangroves.

|  |  | Primary land | | | | | |
| --- | --- | --- | --- | --- | --- | --- | --- |
|  |  | 2010 | | 2050 | | 2100 | |
| Realm | Biome | Min | Max | Min | Max | Min | Max |
| Australasia | 1 | 0.597 | 0.607 | 0.481 | 0.556 | 0.371 | 0.497 |
|  | 2 | 0.298 | 0.326 | 0.193 | 0.310 | 0.088 | 0.296 |
|  | 4 | 0.303 | 0.326 | 0.111 | 0.245 | 0.101 | 0.176 |
|  | 7 | 0.349 | 0.357 | 0.207 | 0.349 | 0.127 | 0.344 |
|  | 8 | 0.269 | 0.286 | 0.057 | 0.283 | 0.034 | 0.280 |
|  | 10 | 0.249 | 0.275 | 0.033 | 0.247 | 0.031 | 0.135 |
|  | 12 | 0.340 | 0.349 | 0.214 | 0.349 | 0.157 | 0.349 |
|  | 13 | 0.320 | 0.325 | 0.211 | 0.322 | 0.175 | 0.321 |
| Afrotropics | 1 | 0.204 | 0.209 | 0.117 | 0.176 | 0.025 | 0.127 |
|  | 2 | 0.211 | 0.224 | 0.049 | 0.219 | 0.000 | 0.201 |
|  | 7 | 0.134 | 0.148 | 0.046 | 0.120 | 0.021 | 0.098 |
|  | 9 | 0.115 | 0.124 | 0.033 | 0.117 | 0.000 | 0.106 |
|  | 10 | 0.039 | 0.045 | 0.020 | 0.034 | 0.000 | 0.034 |
|  | 12 | 0.000 | 0.000 | 0.000 | 0.000 | 0.000 | 0.000 |
|  | 13 | 0.280 | 0.285 | 0.209 | 0.284 | 0.096 | 0.263 |
|  | 14 | 0.000 | 0.000 | 0.000 | 0.000 | 0.000 | 0.000 |
| Indo-Malaya | 1 | 0.458 | 0.487 | 0.322 | 0.370 | 0.196 | 0.286 |
|  | 2 | 0.349 | 0.445 | 0.142 | 0.365 | 0.054 | 0.307 |
|  | 3 | 0.260 | 0.338 | 0.032 | 0.265 | 0.000 | 0.185 |
|  | 4 | 0.232 | 0.277 | 0.105 | 0.225 | 0.083 | 0.166 |
|  | 5 | 0.269 | 0.277 | 0.063 | 0.257 | 0.000 | 0.234 |
|  | 7 | 0.183 | 0.189 | 0.097 | 0.153 | 0.000 | 0.096 |
|  | 9 | 0.711 | 0.727 | 0.000 | 0.587 | 0.000 | 0.580 |
|  | 13 | 0.251 | 0.276 | 0.000 | 0.208 | 0.000 | 0.207 |
|  | 14 | 0.285 | 0.295 | 0.222 | 0.260 | 0.151 | 0.218 |
| Nearctic | 2 | 0.128 | 0.166 | 0.029 | 0.166 | 0.000 | 0.166 |
|  | 3 | 0.443 | 0.469 | 0.272 | 0.469 | 0.137 | 0.468 |
|  | 4 | 0.110 | 0.116 | 0.071 | 0.112 | 0.025 | 0.089 |
|  | 5 | 0.457 | 0.464 | 0.366 | 0.405 | 0.271 | 0.364 |
|  | 6 | 0.816 | 0.824 | 0.720 | 0.806 | 0.572 | 0.776 |
|  | 7 | 0.013 | 0.026 | 0.000 | 0.026 | 0.000 | 0.026 |
|  | 8 | 0.049 | 0.052 | 0.031 | 0.045 | 0.020 | 0.035 |
|  | 11 | 0.887 | 0.887 | 0.873 | 0.879 | 0.857 | 0.876 |
|  | 12 | 0.284 | 0.451 | 0.106 | 0.451 | 0.084 | 0.450 |
|  | 13 | 0.449 | 0.457 | 0.377 | 0.457 | 0.361 | 0.455 |
| Neotropics | 1 | 0.525 | 0.531 | 0.416 | 0.470 | 0.297 | 0.426 |
|  | 2 | 0.249 | 0.287 | 0.092 | 0.277 | 0.028 | 0.264 |
|  | 3 | 0.142 | 0.158 | 0.047 | 0.124 | 0.011 | 0.075 |
|  | 4 | 0.353 | 0.373 | 0.185 | 0.329 | 0.149 | 0.267 |
|  | 7 | 0.260 | 0.282 | 0.141 | 0.265 | 0.085 | 0.251 |
|  | 8 | 0.376 | 0.388 | 0.252 | 0.371 | 0.200 | 0.363 |
|  | 9 | 0.284 | 0.302 | 0.164 | 0.294 | 0.078 | 0.281 |
|  | 10 | 0.477 | 0.480 | 0.419 | 0.477 | 0.358 | 0.474 |
|  | 12 | 0.379 | 0.389 | 0.293 | 0.379 | 0.264 | 0.379 |
|  | 13 | 0.386 | 0.422 | 0.211 | 0.422 | 0.136 | 0.421 |
|  | 14 | 0.234 | 0.261 | 0.148 | 0.243 | 0.074 | 0.232 |
| Palearctic | 1 | 0.348 | 0.360 | 0.133 | 0.272 | 0.034 | 0.190 |
|  | 4 | 0.209 | 0.244 | 0.098 | 0.202 | 0.054 | 0.144 |
|  | 5 | 0.297 | 0.301 | 0.200 | 0.253 | 0.101 | 0.195 |
|  | 6 | 0.628 | 0.641 | 0.473 | 0.581 | 0.257 | 0.499 |
|  | 8 | 0.153 | 0.172 | 0.078 | 0.155 | 0.043 | 0.128 |
|  | 9 | 0.349 | 0.363 | 0.202 | 0.333 | 0.082 | 0.218 |
|  | 10 | 0.244 | 0.254 | 0.175 | 0.224 | 0.123 | 0.176 |
|  | 11 | 0.800 | 0.803 | 0.767 | 0.796 | 0.714 | 0.780 |
|  | 12 | 0.132 | 0.210 | 0.024 | 0.206 | 0.020 | 0.202 |
|  | 13 | 0.298 | 0.305 | 0.280 | 0.291 | 0.260 | 0.277 |
