# Supplementary material for: Global Projections of 21st Century Land-Use Changes in Regions Adjacent to Protected Areas
Source: PLoS One. 2012 Aug 30;7(8):e43714. doi: 10.1371/journal.pone.0043714 (PMC3431375; doi:10.1371/journal.pone.0043714)
Supplement: Table S2 — Projections of the fractional extent of secondary land across 50 km buffer regions that surround the world's Protected Areas. Projections were derived from four land-use scenarios, and are summarized for each Realm/Biome. The table gives the smallest and largest projection of the four scenarios for 2010, 2050 and 2100. See Table S1 for the names of each biome. (DOCX) [file pone.0043714.s002.docx]

Table S2. Projections of the fractional extent of secondary land across 50km buffer regions that surround the world’s Protected Areas. Projections were derived from four land-use scenarios, and are summarized for each Realm/Biome. The table gives the smallest and largest projection of the four scenarios for 2010, 2050 and 2100. See Table S1 for the names of each biome.

|  |  | Secondary land | | | | | |
| --- | --- | --- | --- | --- | --- | --- | --- |
|  |  | 2010 | | 2050 | | 2100 | |
| Realm | Biome | Min | Max | Min | Max | Min | Max |
| Australasia | 1 | 0.150 | 0.160 | 0.172 | 0.268 | 0.241 | 0.351 |
|  | 2 | 0.036 | 0.060 | 0.080 | 0.161 | 0.150 | 0.277 |
|  | 4 | 0.132 | 0.181 | 0.203 | 0.331 | 0.205 | 0.434 |
|  | 7 | 0.067 | 0.073 | 0.066 | 0.339 | 0.060 | 0.619 |
|  | 8 | 0.059 | 0.074 | 0.052 | 0.301 | 0.042 | 0.519 |
|  | 10 | 0.125 | 0.153 | 0.143 | 0.378 | 0.225 | 0.504 |
|  | 12 | 0.060 | 0.067 | 0.061 | 0.294 | 0.063 | 0.426 |
|  | 13 | 0.072 | 0.078 | 0.072 | 0.361 | 0.068 | 0.684 |
| Afrotropics | 1 | 0.407 | 0.476 | 0.407 | 0.656 | 0.411 | 0.710 |
|  | 2 | 0.115 | 0.191 | 0.106 | 0.450 | 0.077 | 0.455 |
|  | 7 | 0.364 | 0.386 | 0.345 | 0.621 | 0.288 | 0.655 |
|  | 9 | 0.446 | 0.469 | 0.326 | 0.746 | 0.316 | 0.747 |
|  | 10 | 0.302 | 0.334 | 0.235 | 0.515 | 0.192 | 0.595 |
|  | 12 | 0.176 | 0.196 | 0.059 | 0.295 | 0.007 | 0.380 |
|  | 13 | 0.065 | 0.080 | 0.056 | 0.304 | 0.122 | 0.427 |
|  | 14 | 0.537 | 0.633 | 0.443 | 0.817 | 0.299 | 0.817 |
| Indo-Malaya | 1 | 0.109 | 0.138 | 0.221 | 0.281 | 0.310 | 0.410 |
|  | 2 | 0.062 | 0.156 | 0.134 | 0.317 | 0.240 | 0.376 |
|  | 3 | 0.215 | 0.312 | 0.260 | 0.522 | 0.338 | 0.537 |
|  | 4 | 0.330 | 0.381 | 0.308 | 0.508 | 0.367 | 0.602 |
|  | 5 | 0.363 | 0.373 | 0.315 | 0.590 | 0.363 | 0.681 |
|  | 7 | 0.348 | 0.360 | 0.257 | 0.730 | 0.297 | 0.821 |
|  | 9 | 0.000 | 0.011 | 0.104 | 0.716 | 0.124 | 0.749 |
|  | 13 | 0.175 | 0.199 | 0.230 | 0.451 | 0.249 | 0.498 |
|  | 14 | 0.089 | 0.103 | 0.102 | 0.173 | 0.134 | 0.246 |
| Nearctic | 2 | 0.047 | 0.077 | 0.053 | 0.265 | 0.057 | 0.328 |
|  | 3 | 0.042 | 0.049 | 0.044 | 0.166 | 0.045 | 0.286 |
|  | 4 | 0.505 | 0.524 | 0.423 | 0.647 | 0.504 | 0.735 |
|  | 5 | 0.311 | 0.315 | 0.334 | 0.397 | 0.400 | 0.494 |
|  | 6 | 0.094 | 0.105 | 0.099 | 0.214 | 0.126 | 0.362 |
|  | 7 | 0.108 | 0.124 | 0.088 | 0.151 | 0.127 | 0.173 |
|  | 8 | 0.078 | 0.092 | 0.069 | 0.185 | 0.086 | 0.240 |
|  | 11 | 0.006 | 0.007 | 0.007 | 0.020 | 0.016 | 0.035 |
|  | 12 | 0.086 | 0.255 | 0.098 | 0.376 | 0.098 | 0.441 |
|  | 13 | 0.076 | 0.081 | 0.082 | 0.131 | 0.080 | 0.215 |
| Neotropics | 1 | 0.229 | 0.249 | 0.301 | 0.377 | 0.364 | 0.444 |
|  | 2 | 0.219 | 0.244 | 0.237 | 0.358 | 0.244 | 0.480 |
|  | 3 | 0.348 | 0.388 | 0.351 | 0.528 | 0.334 | 0.630 |
|  | 4 | 0.267 | 0.290 | 0.301 | 0.500 | 0.380 | 0.542 |
|  | 7 | 0.178 | 0.210 | 0.200 | 0.382 | 0.218 | 0.520 |
|  | 8 | 0.130 | 0.139 | 0.157 | 0.228 | 0.169 | 0.415 |
|  | 9 | 0.151 | 0.167 | 0.161 | 0.350 | 0.153 | 0.533 |
|  | 10 | 0.292 | 0.299 | 0.290 | 0.324 | 0.287 | 0.388 |
|  | 12 | 0.148 | 0.167 | 0.144 | 0.279 | 0.168 | 0.355 |
|  | 13 | 0.183 | 0.198 | 0.176 | 0.350 | 0.186 | 0.404 |
|  | 14 | 0.179 | 0.198 | 0.211 | 0.276 | 0.211 | 0.307 |
| Palearctic | 1 | 0.049 | 0.063 | 0.145 | 0.267 | 0.232 | 0.439 |
|  | 4 | 0.235 | 0.266 | 0.277 | 0.393 | 0.313 | 0.467 |
|  | 5 | 0.315 | 0.329 | 0.356 | 0.478 | 0.413 | 0.597 |
|  | 6 | 0.272 | 0.289 | 0.331 | 0.441 | 0.413 | 0.655 |
|  | 8 | 0.090 | 0.104 | 0.111 | 0.308 | 0.117 | 0.357 |
|  | 9 | 0.148 | 0.217 | 0.187 | 0.470 | 0.206 | 0.622 |
|  | 10 | 0.109 | 0.126 | 0.138 | 0.278 | 0.162 | 0.326 |
|  | 11 | 0.091 | 0.094 | 0.098 | 0.127 | 0.114 | 0.181 |
|  | 12 | 0.163 | 0.248 | 0.186 | 0.385 | 0.211 | 0.398 |
|  | 13 | 0.255 | 0.263 | 0.265 | 0.311 | 0.269 | 0.328 |
