# Supplementary material for: Global Projections of 21st Century Land-Use Changes in Regions Adjacent to Protected Areas
Source: PLoS One. 2012 Aug 30;7(8):e43714. doi: 10.1371/journal.pone.0043714 (PMC3431375; doi:10.1371/journal.pone.0043714)
Supplement: Table S3 — Projections of the fractional extent of pasture land across 50 km buffer regions that surround the world's Protected Areas. Projections were derived from four land-use scenarios, and are summarized for each Realm/Biome. The table gives the smallest and largest projection of the four scenarios for 2010, 2050 and 2100. See Table S1 for the names of each biome. (DOCX) [file pone.0043714.s003.docx]

Table S3. Projections of the fractional extent of pasture land across 50km buffer regions that surround the world’s Protected Areas. Projections were derived from four land-use scenarios, and are summarized for each Realm/Biome. The table gives the smallest and largest projection of the four scenarios for 2010, 2050 and 2100. See Table S1 for the names of each biome.

|  |  | Pasture land | | | | | |
| --- | --- | --- | --- | --- | --- | --- | --- |
|  |  | 2010 | | 2050 | | 2100 | |
| Realm | Biome | Min | Max | Min | Max | Min | Max |
| Australasia | 1 | 0.081 | 0.083 | 0.043 | 0.107 | 0.036 | 0.128 |
|  | 2 | 0.087 | 0.090 | 0.062 | 0.092 | 0.057 | 0.085 |
|  | 4 | 0.245 | 0.260 | 0.134 | 0.314 | 0.028 | 0.313 |
|  | 7 | 0.474 | 0.486 | 0.300 | 0.622 | 0.035 | 0.687 |
|  | 8 | 0.431 | 0.454 | 0.278 | 0.670 | 0.045 | 0.699 |
|  | 10 | 0.569 | 0.577 | 0.465 | 0.630 | 0.240 | 0.642 |
|  | 12 | 0.326 | 0.343 | 0.171 | 0.425 | 0.027 | 0.472 |
|  | 13 | 0.585 | 0.596 | 0.415 | 0.653 | 0.121 | 0.744 |
| Afrotropics | 1 | 0.177 | 0.207 | 0.079 | 0.241 | 0.060 | 0.265 |
|  | 2 | 0.533 | 0.604 | 0.305 | 0.706 | 0.302 | 0.739 |
|  | 7 | 0.343 | 0.356 | 0.132 | 0.415 | 0.079 | 0.445 |
|  | 9 | 0.319 | 0.329 | 0.106 | 0.369 | 0.071 | 0.380 |
|  | 10 | 0.419 | 0.447 | 0.237 | 0.507 | 0.129 | 0.529 |
|  | 12 | 0.633 | 0.655 | 0.518 | 0.769 | 0.415 | 0.819 |
|  | 13 | 0.590 | 0.609 | 0.359 | 0.678 | 0.242 | 0.700 |
|  | 14 | 0.130 | 0.168 | 0.000 | 0.182 | 0.000 | 0.228 |
| Indo-Malaya | 1 | 0.078 | 0.080 | 0.058 | 0.081 | 0.062 | 0.083 |
|  | 2 | 0.033 | 0.036 | 0.022 | 0.032 | 0.010 | 0.035 |
|  | 3 | 0.055 | 0.057 | 0.041 | 0.058 | 0.025 | 0.058 |
|  | 4 | 0.075 | 0.078 | 0.060 | 0.077 | 0.044 | 0.079 |
|  | 5 | 0.125 | 0.129 | 0.089 | 0.126 | 0.067 | 0.126 |
|  | 7 | 0.033 | 0.035 | 0.019 | 0.032 | 0.006 | 0.032 |
|  | 9 | 0.023 | 0.028 | 0.005 | 0.043 | 0.004 | 0.045 |
|  | 13 | 0.040 | 0.046 | 0.014 | 0.050 | 0.007 | 0.052 |
|  | 14 | 0.014 | 0.015 | 0.009 | 0.015 | 0.006 | 0.015 |
| Nearctic | 2 | 0.324 | 0.338 | 0.130 | 0.342 | 0.076 | 0.357 |
|  | 3 | 0.364 | 0.383 | 0.277 | 0.474 | 0.177 | 0.577 |
|  | 4 | 0.084 | 0.091 | 0.049 | 0.179 | 0.049 | 0.101 |
|  | 5 | 0.100 | 0.103 | 0.086 | 0.161 | 0.087 | 0.101 |
|  | 6 | 0.004 | 0.008 | 0.001 | 0.023 | 0.001 | 0.006 |
|  | 7 | 0.223 | 0.233 | 0.211 | 0.259 | 0.151 | 0.237 |
|  | 8 | 0.368 | 0.381 | 0.355 | 0.386 | 0.284 | 0.364 |
|  | 11 | 0.001 | 0.001 | 0.001 | 0.010 | 0.001 | 0.002 |
|  | 12 | 0.194 | 0.202 | 0.192 | 0.231 | 0.126 | 0.214 |
|  | 13 | 0.385 | 0.391 | 0.382 | 0.425 | 0.318 | 0.393 |
| Neotropics | 1 | 0.138 | 0.150 | 0.092 | 0.161 | 0.047 | 0.166 |
|  | 2 | 0.308 | 0.327 | 0.222 | 0.377 | 0.124 | 0.408 |
|  | 3 | 0.229 | 0.253 | 0.157 | 0.312 | 0.076 | 0.339 |
|  | 4 | 0.155 | 0.167 | 0.117 | 0.225 | 0.086 | 0.254 |
|  | 7 | 0.414 | 0.447 | 0.269 | 0.484 | 0.090 | 0.495 |
|  | 8 | 0.401 | 0.419 | 0.332 | 0.509 | 0.149 | 0.543 |
|  | 9 | 0.437 | 0.458 | 0.299 | 0.507 | 0.081 | 0.518 |
|  | 10 | 0.204 | 0.210 | 0.183 | 0.236 | 0.120 | 0.246 |
|  | 12 | 0.309 | 0.318 | 0.276 | 0.321 | 0.201 | 0.316 |
|  | 13 | 0.256 | 0.280 | 0.137 | 0.399 | 0.076 | 0.466 |
|  | 14 | 0.120 | 0.129 | 0.065 | 0.141 | 0.044 | 0.154 |
| Palearctic | 1 | 0.348 | 0.357 | 0.276 | 0.350 | 0.292 | 0.383 |
|  | 4 | 0.159 | 0.173 | 0.127 | 0.177 | 0.097 | 0.182 |
|  | 5 | 0.245 | 0.253 | 0.203 | 0.250 | 0.209 | 0.252 |
|  | 6 | 0.019 | 0.028 | 0.003 | 0.028 | 0.003 | 0.028 |
|  | 8 | 0.401 | 0.413 | 0.321 | 0.413 | 0.276 | 0.423 |
|  | 9 | 0.186 | 0.228 | 0.100 | 0.223 | 0.108 | 0.230 |
|  | 10 | 0.547 | 0.556 | 0.454 | 0.549 | 0.461 | 0.567 |
|  | 11 | 0.001 | 0.002 | 0.000 | 0.002 | 0.000 | 0.002 |
|  | 12 | 0.204 | 0.211 | 0.204 | 0.231 | 0.171 | 0.254 |
|  | 13 | 0.381 | 0.384 | 0.347 | 0.382 | 0.336 | 0.387 |

Table S4. Projections of the fractional extent of crop land across 50km buffer regions that surround the world’s Protected Areas. Projections were derived from four land-use scenarios, and are summarized for each Realm/Biome. The table gives the smallest and largest projection of the four scenarios for 2010, 2050 and 2100. See Table S1 for the names of each biome.

|  |  | Crop land | | | | | |
| --- | --- | --- | --- | --- | --- | --- | --- |
|  |  | 2010 |  | 2050 | | 2100 | |
| Realm | Biome | Min | Max | Min | Max | Min | Max |
| Australasia | 1 | 0.058 | 0.063 | 0.044 | 0.096 | 0.035 | 0.112 |
|  | 2 | 0.206 | 0.209 | 0.175 | 0.211 | 0.154 | 0.213 |
|  | 4 | 0.148 | 0.207 | 0.103 | 0.277 | 0.091 | 0.284 |
|  | 7 | 0.056 | 0.063 | 0.039 | 0.146 | 0.035 | 0.226 |
|  | 8 | 0.185 | 0.204 | 0.129 | 0.228 | 0.115 | 0.257 |
|  | 10 | 0.002 | 0.029 | 0.001 | 0.098 | 0.001 | 0.134 |
|  | 12 | 0.214 | 0.219 | 0.149 | 0.258 | 0.133 | 0.306 |
|  | 13 | 0.008 | 0.008 | 0.005 | 0.010 | 0.005 | 0.020 |
| Afrotropics | 1 | 0.107 | 0.149 | 0.057 | 0.224 | 0.052 | 0.245 |
|  | 2 | 0.018 | 0.035 | 0.005 | 0.097 | 0.005 | 0.141 |
|  | 7 | 0.111 | 0.126 | 0.094 | 0.218 | 0.086 | 0.242 |
|  | 9 | 0.067 | 0.079 | 0.057 | 0.224 | 0.052 | 0.242 |
|  | 10 | 0.180 | 0.205 | 0.146 | 0.271 | 0.133 | 0.302 |
|  | 12 | 0.100 | 0.106 | 0.091 | 0.141 | 0.083 | 0.148 |
|  | 13 | 0.022 | 0.024 | 0.021 | 0.033 | 0.019 | 0.043 |
|  | 14 | 0.054 | 0.106 | 0.000 | 0.159 | 0.000 | 0.199 |
| Indo-Malaya | 1 | 0.272 | 0.277 | 0.242 | 0.315 | 0.193 | 0.331 |
|  | 2 | 0.435 | 0.438 | 0.447 | 0.486 | 0.396 | 0.557 |
|  | 3 | 0.364 | 0.390 | 0.278 | 0.479 | 0.246 | 0.463 |
|  | 4 | 0.303 | 0.310 | 0.202 | 0.440 | 0.176 | 0.446 |
|  | 5 | 0.211 | 0.215 | 0.150 | 0.351 | 0.135 | 0.356 |
|  | 7 | 0.407 | 0.411 | 0.096 | 0.557 | 0.070 | 0.568 |
|  | 9 | 0.184 | 0.198 | 0.179 | 0.280 | 0.145 | 0.431 |
|  | 13 | 0.479 | 0.482 | 0.475 | 0.530 | 0.427 | 0.549 |
|  | 14 | 0.192 | 0.197 | 0.165 | 0.214 | 0.136 | 0.220 |
| Nearctic | 2 | 0.211 | 0.218 | 0.202 | 0.276 | 0.199 | 0.312 |
|  | 3 | 0.115 | 0.120 | 0.110 | 0.162 | 0.108 | 0.190 |
|  | 4 | 0.197 | 0.214 | 0.149 | 0.222 | 0.113 | 0.269 |
|  | 5 | 0.058 | 0.064 | 0.049 | 0.112 | 0.042 | 0.147 |
|  | 6 | 0.009 | 0.013 | 0.001 | 0.019 | 0.001 | 0.042 |
|  | 7 | 0.296 | 0.304 | 0.279 | 0.310 | 0.258 | 0.313 |
|  | 8 | 0.458 | 0.482 | 0.395 | 0.489 | 0.350 | 0.555 |
|  | 11 | 0.000 | 0.000 | 0.000 | 0.001 | 0.000 | 0.001 |
|  | 12 | 0.095 | 0.097 | 0.082 | 0.236 | 0.089 | 0.354 |
|  | 13 | 0.056 | 0.058 | 0.055 | 0.102 | 0.053 | 0.118 |
| Neotropics | 1 | 0.058 | 0.072 | 0.036 | 0.099 | 0.028 | 0.114 |
|  | 2 | 0.119 | 0.135 | 0.098 | 0.188 | 0.090 | 0.235 |
|  | 3 | 0.197 | 0.229 | 0.143 | 0.286 | 0.124 | 0.317 |
|  | 4 | 0.023 | 0.029 | 0.014 | 0.054 | 0.011 | 0.084 |
|  | 7 | 0.082 | 0.101 | 0.053 | 0.137 | 0.042 | 0.157 |
|  | 8 | 0.036 | 0.041 | 0.034 | 0.056 | 0.033 | 0.074 |
|  | 9 | 0.045 | 0.054 | 0.043 | 0.103 | 0.039 | 0.130 |
|  | 10 | 0.012 | 0.016 | 0.010 | 0.029 | 0.010 | 0.041 |
|  | 12 | 0.111 | 0.126 | 0.086 | 0.143 | 0.076 | 0.165 |
|  | 13 | 0.089 | 0.103 | 0.085 | 0.158 | 0.083 | 0.213 |
|  | 14 | 0.058 | 0.072 | 0.051 | 0.093 | 0.048 | 0.108 |
| Palearctic | 1 | 0.228 | 0.233 | 0.223 | 0.317 | 0.217 | 0.274 |
|  | 4 | 0.304 | 0.315 | 0.251 | 0.327 | 0.215 | 0.386 |
|  | 5 | 0.100 | 0.104 | 0.089 | 0.112 | 0.063 | 0.133 |
|  | 6 | 0.021 | 0.027 | 0.004 | 0.031 | 0.003 | 0.049 |
|  | 8 | 0.299 | 0.310 | 0.193 | 0.335 | 0.170 | 0.393 |
|  | 9 | 0.205 | 0.224 | 0.186 | 0.252 | 0.146 | 0.286 |
|  | 10 | 0.056 | 0.057 | 0.056 | 0.071 | 0.055 | 0.079 |
|  | 11 | 0.001 | 0.001 | 0.001 | 0.002 | 0.001 | 0.002 |
|  | 12 | 0.307 | 0.318 | 0.263 | 0.310 | 0.221 | 0.337 |
|  | 13 | 0.040 | 0.041 | 0.041 | 0.051 | 0.037 | 0.056 |
